# Development of Polyhydroxyalkanoate-Based Polyurethane with Water-Thermal Response Shape-Memory Behavior as New 3D Elastomers Scaffolds

**DOI:** 10.3390/polym11061030

**Published:** 2019-06-11

**Authors:** Cai Wang, Han Wang, Faxing Zou, Shaojun Chen, Yiping Wang

**Affiliations:** 1 Guangdong Research Center for Interfacial Engineering of Functional Materials, Shenzhen Key Laboratory of Polymer Science and Technology, Shenzhen Key Laboratory of Special Functional Materials, Nanshan District Key Lab for Biopolymers and Safety Evaluation, College of Materials Science and Engineering, Shenzhen University, Shenzhen 518060, China; whxtbh@163.com (C.W.); esmee_wang@163.com (H.W.); 2160120419@email.szu.edu.cn (F.Z.); 2 Key Laboratory of Optoelectronic Devices and Systems of Ministry of Education and Guangdong Province, College of Optoelectronic Engineering, Shenzhen University, Shenzhen 518060, China

**Keywords:** polyhydroxyalkanoate, polyurethane, water-thermal responsive, shape-memory, scaffolds

## Abstract

In this study, we report the synthesis of a novel bio-based material from polyhydroxyalkanoate (PHA) with good shape-memory effect (SME) and rapid recovery. In this PHA-based polyurethane (PHP), telechelic-hydroxylated polyhydroxyalkanoate (PHA-diols) and polyethylene glycol (PEG) were used as soft segments, providing thermo-responsive domains and water-responsive regions, respectively. Thus, PHP possesses good thermal-responsive SME, such as high shape fixing (>99%) and shape recovery ratio (>90%). Upon immersing in water, the storage modulus of PHP decreased considerably owing to disruption of hydrogen bonds in the PHP matrix. Their water-responsive SME is also suitable for rapid shape recovery (less than 10 s). Furthermore, these outstanding properties can trigger shape-morphing, enabling self-folding and self-expansion of shapes into three-dimensional (3D) scaffolds for potential biomedical applications.

## 1. Introduction

As one of the most promising intelligent materials, shape-memory polymers (SMPs) can stabilize their temporary shape and return to their original shape when exposed to external stimuli such as heat, light, pH, and magnetism [1,2,3,4,5]. Therefore, SMPs have attracted increasing attention and have shown promise of application in many fields, such as drug delivery, diagnostics, smart optical systems, biosensors, smart textiles, and artificial tissue engineering [4,6,7,8]. Traditional SMPs are thermo-responsive polymers, the shapes of which are deformed at high temperature, usually above the glass transition (*T*_g_) or crystal melting temperature (*T*_m_), and subsequently frozen to a temporary shape at low temperature [9,10]. Thermo-responsive SMPs can be used in most of the above applications as their shape recovery is generally triggered by heating above a transition temperature. However, if the temporary shape is complex or the operation environment is not suitable for direct contact, the commonly used deformation method cannot be optimally used, which considerably restricts the potential applications of SMPs.

Recent advances in SMPs have resulted in multiple shape-memory effects (SMEs) and responses [11,12,13]. Among all stimuli-responsive materials, water-responsive SMPs have always received significant interest due to their remarkable advantages, such as convenience of responding and biocompatibility. Thus, SMPs triggered by water, the safest and most abundant solvent in nature, may be ideal candidates for smart biological materials. However, most of the previous water-responsive SMPs show relatively slow responsivity [14,15,16,17]. In fact, preparing water-responsive SMPs with fast responsivity is beneficial for many fields, including sensors, artificial muscles, and fast actuating devices [18]. In addition, the biocompatibility of SMPs has to be improved further before they can be used for various biomedical applications [19].

In particular, water-responsive SMPs have been shown to be useful for biomedical tissue engineering [20]. Complicated tissue defects have increased the demand for the construction of substitutes of diverse shapes. SMP-based structures have been shown to evolve into bio-inspired three-dimensional (3D) shapes from two-dimensional (2D) structures. The self-folding of the structure is usually controlled by the hydrophilic–hydrophobic effect or temperature-sensitive effect in a simulated biological environment. The ability to control shape-morphing of a material system using water-responsive SMPs may emerge as a new general strategy for programming complex soft structures.

The limitations of current water-triggered SMPs highlighted the need for new material and process design strategies that may improve recovery ratios and enhance recovery speed. Therefore, we proposed another type of water-responsive SMP using polyhydroxyalkanoate (PHA), a bio-based polymer, which is well known for its biodegradability and biocompatibility in biomedical applications [21,22]. In particular, self-folding and self-expandable 3D stents have been developed using modified intelligent manufacturing techniques, such as the origami technique, photolithography, dip-dyeing, and 3D printing [23,24]. In contrast, water-responsive shape-memory materials with rapid responsivity were prepared using simple and convenient solution casting and particulate leaching methods. This study might provide extensive technical guidance for the development of new SMPs.

## 2. Materials and Methods

### 2.1. Materials

The poly(hydroxyalkanoate) powders (PHA, 3.2 × 10^5^ g/mol) were purchased from TianAn Biologic Materials, Ningbo, China. Telechelic-hydroxylated poly(hydroxyalkanoate)s (PHA-diols) were synthesized by transesterification with butanediol, which can be used as macromonomers for the synthesis of block copolymers. The telechelic-hydroxylated polyhydroxyalkanoate (PHA-diols; 1672 g/mol, determined using NMR spectroscopy) used in this study were derived from depolymerization of the natural PHA polymer powders produced by *Penicillium* sp. Hexamethylene diisocyanate (HDI, Aladdin, Shanghai, China) was used as received. Polyethylene glycol (PEG) (Mw = 1000, obtained from Aladdin) was dehydrated at 100 °C for 4 h under vacuum. Chloroform for polymer synthesis was dried over calcium chloride and distilled prior to use. Butanediol (BDO), stannous octoate, and p-toluene sulfonic acid (PTSA) were purchased from Aladdin. All other chemicals were of reagent grade.

### 2.2. PEG–HDI–PHA Synthesis

**Figure 1:**
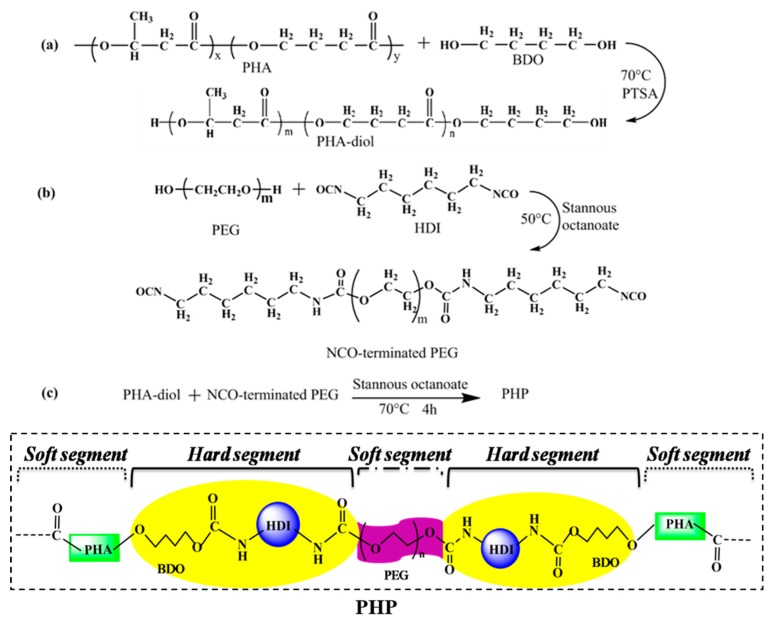
Illustration for the synthetic process of (**a**) PHA-diol, (**b**) the prepolymer(NCO-terminated PEG), (**c**) polyhydroxyalkanoate (PHA)-based polyurethane (PHP) and their structure.

The superior bioelastomers (PEG–HDI–PHA, PHP) were synthesized by bridging hexamethylenediisocyanate (HDI) with PEG and PHA using a facile curing process. The synthetic procedures are illustrated in Figure 1. For polymerization, the PHA polymer was first purified via recrystallization, dissolved in trichloromethane (solvent) at room temperature under a nitrogen atmosphere, reacted with butanediol for transesterification in the presence of p-toluene sulphonic acid (PTSA) for 4 h at 70 °C, and further purified via extraction, precipitation, and filtration, as shown in Figure 1a. The following is the detailed procedure used for preparing PHP. After PEG-1000 (1.0 g) and HDI (0.32 g) were added to a 100 mL three-neck round flask filled with N_2_ gas, 10 mL dimethyl formamide (DMF) solvent and 0.03% stannous octoate catalyst were added to the reaction mixture. The reaction was mechanically stirred at 50 °C for 4 h, as shown in Figure 1b. Next, PHA-diol (0.68 g) was added to the prepolymer (NCO-terminated PEG), and the reaction was mechanically stirred at 70 °C for 4 h (Figure 1c). Then, the mixture was poured into a Teflon dish, which was dried at 80 °C for 48 h to remove the residual solvent. The structure of the prepared PHP is shown in Figure 1c, which is a typical multiblock polyurethane. The PHP was thus designed to consist of BDO and HDI units in their hard segments, and PEG and PHA units in their soft segments (Figure 1c). For comparison, we prepared three samples, PHA, PEG–HDI, and PEG–HDI–PHA.

### 2.3. Characterization

Fourier transform infrared (FT-IR) spectrometry (Nicolet 6700, ThermoFisher, Waltham, MA, USA) and laser Raman spectrometry (LRS) (Thermo-Fisher Smart Raman DXR, a 532 nm laser at 10% intension, and samples were exposed for 30 s) were used to detect the chemical composition of the polymers. The crystallinity of PHP films was investigated in the scan range from 2*θ* = 10° to 50° using X-ray diffraction (XRD) with a scanning rate of 10° min^−1^. Differential scanning calorimetry (DSC) was performed using the Perkin Elmer Pyris 6 equipment (Wilmington, NC, USA), and the glass-transition temperature (*T*_g_) or the melting temperature (*T*_m_) was determined. 

The microstructures of PHP films and porous scaffold surfaces were analyzed using a scanning electron microscope (SEM; S4800, Hitachi, Tokyo, Japan). All the surfaces were coated with Au before measurement. 

The dynamic mechanical properties were measured on a dynamic thermo-mechanical analysis (DMA) Q800 instrument (TA Instruments, Wilmington, NC, USA) using the tensile mode at a constant frequency of 1.0 Hz from −60 to 100 °C at a heating rate of 3 °C/min. The test specimen dimensions were 25 mm × 4 mm. To determine the tensile properties of the PHP films in the wet state, the samples were swollen in pure water at room temperature for a certainduration and further cleaned gently with filter papers prior to the test.

Tensile and compressive tests were performed using a universal testing instrument (AGIC 50KN, Shimadzu, Japan) with a 500 N load cell for tension and 2000 N for compression. The specimens were cut into 20 mm × 5 mm (length × width) samples, and the tensile properties were measured at a constant speed of 50 mm/min at 25 °C. The cylinder samples with height of 20 mm and diameter of 15 mm were used for compression tests. The compressive strain was estimated as *h*/*h*_0_, where *h* is the height under compression and *h*_0_ is the original height. The compressive rate was 5 mm/min. Compression cycle tests were performed in subsequent trials immediately after the initial loading at the same velocity of 5 mm/min. Each sample was tested five times with identical specimens, and their average mechanical properties were reported.

### 2.4. Shape-Memory Properties

The shape-memory behaviors were investigated using a TA Instrument’s DMA Q800 (tension clamp, controlled-force mode). The films were cut into rectangular specimens (10 mm × 5 mm × 0.5 mm). Typically, the process involved the following steps: (1) the rectangular sample was heated and held at 100 °C for 20 min; (2) the sample was uniaxially elongated by a ramping force from 0.00 N to 0.15 N at a rate of 0.05 N min^−1^ and kept fully stretched at a fixed strain; (3) the sample was cooled rapidly at a rate of 30 °C min^−1^ to a low temperature at a fixed strain; (4) unloading of the extra force from 0.15 N to 0 N was performed at the rate of 0.05 N min^−1^; (5) the sample was reheated to 100 °C at a rate of 10 °C min^−1^ and held at 100 °C for 40 min. During this process, DMA curves showing the strain, temperature, and time were recorded for future analysis.

## 3. Results and Discussion

### 3.1. Structural Analysis

**Figure 2:**
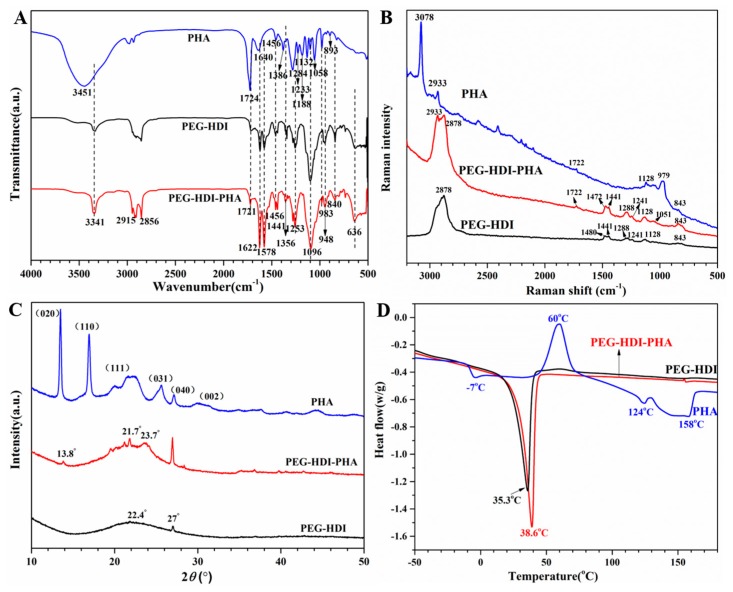
Characterization of the synthesized PHA-based polyurethane: (**A**) FT-IR spectra and (**B**) Raman spectra; (**C**) XRD analysis and (**D**) the second DSC heating curves.

FT-IR and Raman spectrum were first used to confirm the molecular structure of the PHA-based polyurethane, as shown in Figure 2A,B. In the FT-IR spectrum, the characteristic –NCO absorption peak at 2260 cm^−1^ was absent, indicating the complete reaction of isocyanate with a hydroxyl group. The presence of N–H and C=O vibrations at approximately 3341 cm^−1^ and 1721 cm^−1^, respectively, were indicative of hydrogen bonding between the N–H and C=O groups in urethane, which is consistent with the existing literature [25,26]. In addition, C=O stretching vibration of PHA was observed at approximately 1724 cm^−1^. Each peak with fixed wavelengths is given in Table S1. The Raman spectrum of PHP is shown in Figure 2B, which showed stretching vibrations of C=O at 1722 cm^−1^, indicative of PHA skeletal deformation. There was no peak at 1722 cm^−1^ in PEG–HDI, which is consistent with the FT-IR results.

To study the effect of PHA content on the crystallinity of the PHA-based polyurethane, the crystal structures were characterized using XRD. The XRD patterns of the PHP films are shown in Figure 2C. PHA exhibited characteristic diffraction peaks at 2θ values of 14.6°, 16.4°, 22.6°, 25.5°, 26.7°, and 30.3°, which were assigned to the (020), (110), (111), (031), (040), and (002) planes, respectively, confirming the crystalline structure of PHA [27]. The diffractograms showed that PEG-HDI is basically amorphous, with a broad diffraction band at 2θ = 22.4°, a small sharp diffraction band at 2θ = 27°, and no other defined diffraction peaks. However, PHP showed the existence of a crystalline zone in the polymer chain. The intensity of crystallinity was better in PHP. This was because of the strong crystallizing power of the PHA macromolecule. The thermal properties of PHA, PEG–HDI, and PEG–HDI–PHA are shown in Figure 2D. The second DSC heating curves showed that *T*_m_ and the melting range of PEG–HDI–PHA was different from that of PEG–HDI. These variations in *T*_m_ and melting range were caused by the increase in crystallinity of the soft segment of PHA, which facilitated crystal formation of the hard segment as previously reported [28]. The increase in the degree of crystallinity was lesser for PEG–HDI–PHA than for PEG–HDI, which increased the *T*_m_ of the PHA-based polyurethane. Possibly there is an increase in the extent of hydrogen bonding, which can act as the physical crosslinker. These results were consistent with the change in trend observed in the XRD measurements.

### 3.2. Fabrication of PEG–HDI–PHA Elastomers into 3D Scaffolds

**Figure 3:**
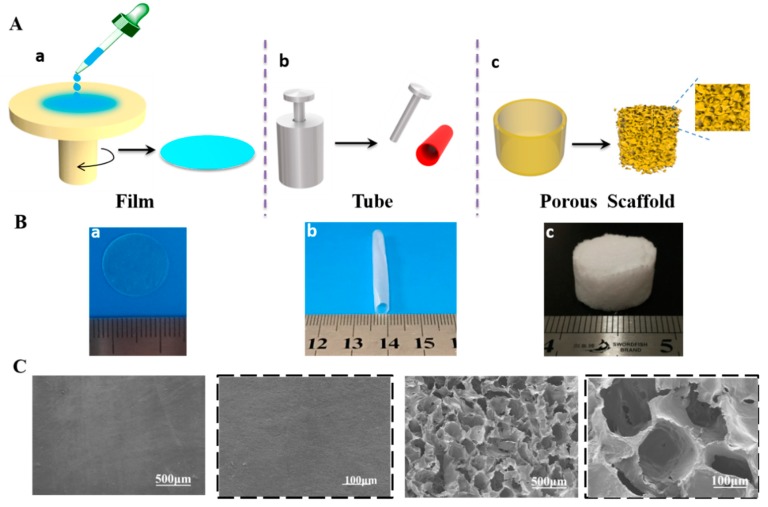
(**A**) Scheme for preparing polymer film, tube, and scaffold using the solvent-free method. (**B**) Photos of the prepared film, tube, and scaffold. (**C**) SEM of the prepared film and scaffold.

Figure 3 shows the successful fabrication of the membrane, tube, and porous scaffold from the synthesized PHP. The membrane of the PHA-based polyurethane was initially prepared using the spin-coating method. A uniform and transparent membrane (Figure 3(Ba)) was obtained after the spin-coating procedure and liquid–solid transformation at room temperature (Figure 3(Aa)). This membrane might be used for soft tissue engineering applications such as cardiac patch, tympanic membrane, and cartilage remodeling [29,30,31]. The tubular form, which might be utilized for preparing vessel constructs [32,33] and nerve conduits [34], is obtained simply by filling the mixture into a modular mold (Figure 3(Ab)). As shown in Figure 3(Bb), the resultant tube had homogeneous wall which was 4 mm in diameter and 0.5 mm thick, with excellent flexibility. This property rendered the PHP a potential candidate in vascular and nerve tissue engineering. In addition, a porous scaffold (Figure 3(Ac,Bc)) was fabricated in a teflon mold in a moist ambience using a particulate-leaching method, which is shown in the supplement document. Such substrates with porous structure, analogous to the natural extracellular matrix (ECM), can be an effective substitute for exchanging medium and allowing transmission of nutrition, which consequently regulates cell behavior and tissue regeneration [35,36]. These shapes indicated that the PHP elastomers can be easily processed and made into corresponding shapes for different potential biomedical applications. Figure 3C shows the cross-sectional SEM images of the PHP films and the porous scaffold, respectively. The surface of the PHP film was smooth. The amplified image clearly shows the smooth surface, demonstrating that PHA had been successfully molded into the novel PHP polymer elastomers. The PEG–HDI–PHA elastomer scaffold showed honeycomb-like porous structures. The pore size was approximately 150 μm (from amplified image), which might support cell growth [37]. 

### 3.3. Mechanical Properties

**Figure 4:**
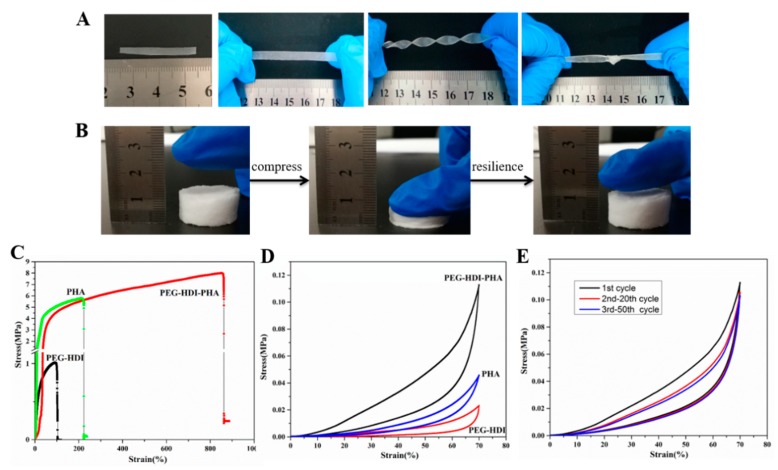
(**A**) Photographs of PHP films showing original length and length after being stretched at λ = 3, and their ability to withstand large stretching, twisting, and knotting; (**B**) Photographs of PHP porous scaffold showing compressibility and resilience; (**C**) Tensile stress–strain curves of PHP films; (**D**) Compressive stress–strain curves of PHP porous scaffold; (**E**) The relaxation cycles to the same polyethylene glycol (PEG)–hexamethylenediisocyanate (HDI)–polyhydroxyalkanoate (PHA) for 50 cycles under a compression of 70%.

Mechanical properties, including tensile strength and stretchability of the pristine PHA, PEG–HDI, and PEG–HDI–PHA films were evaluated systematically. The mechanical properties of the PEG–HDI–PHA porous scaffold were also studied. The mechanical properties of PHP are shown in Figure 4. The synthesized PHA-based polyurethane exhibits good stretching and compression performance. The original length of the film was approximately 30 mm (see Figure 4A). Considering the mechanical properties of PHP membranes, complex deformations such as stretching, revolving, and knotting could be performed on all PHP membranes without fracture (Figure 4A), demonstrating their excellent flexibility and stretchability. Figure 4B shows that the porous scaffold for PHP possesses good compressive resilience under an external force. The elongation at rupture for pristine PHA film reached 220% at a tensile stress of 6.2 MPa. The elongation at rupture for PEG–HDI films reached 100% at a tensile stress of 1.1 MPa. In contrast, the PEG–HDI–PHA films showed increased mechanical properties for the elongation at rupture (860%) and tensile strength (8.3 MPa) (see Figure 4C). In Figure 4C, the stress–strain curve for PHP depicts an initial non-linear regime at about 20–40 (%). The PHP polymer material presented as soft and tough because of its high deformation/low force characteristic. Compared to PHA and PEG–HDI, the PEG–HDI–PHA porous scaffold had the best compression-resilience property at the same 70% compression strain, according to the curve area in Figure 4D. Cyclic loading measurements were performed to better understand the recovery performance of the PEG–HDI–PHA porous scaffold. As shown in Figure 4E, the hysteresis loop for the 50th cycle almost recovered to its 30th cycle state, which was different form the 1st cycle. Owing to dissipation of energy, the recovery rate for the original state decreased, although it stabilized with time. The excellent mechanical performance of PHA-based polyurethane ensures good stretchability and compressibility of the hydrogen bonds and chemical cross-linking in the PHP polymer network.

### 3.4. Water/Thermal Dual-Responsive Shape-Memory Properties

**Figure 5:**
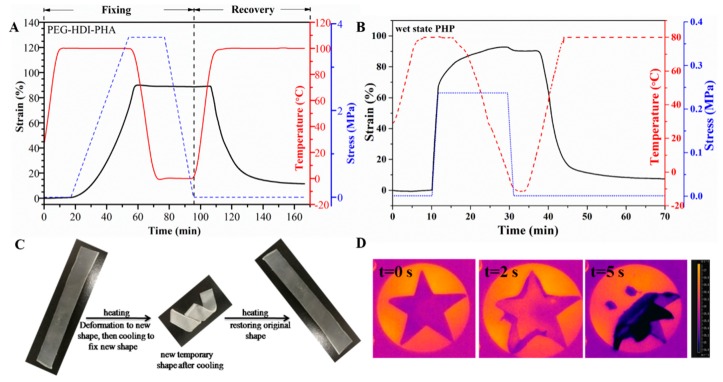
Dual-shape memory cycles of samples. (**A**) PHP in dry state and (**B**) wet state. (**C**) The shape-memory effect of PEG–HDI–PHA; initial state; deformed state (deformed at 80 °C, and fixed at −10 °C); recovered state after being placed in 80 °C bake oven for 10 min. (**D**) The shape-deformed state after being soaked in water for 5 s at the room temperature (27 °C).

To directly study the shape-memory properties of the PHA-based polyurethane, sample PHP was selected as the reference. Figure 5A shows that the PHP sample could be deformed to approximately 95% strain when the sample was heated to 70 °C. After cooling below −4 °C, more than 99% of the strain could be fixed on the first cycle. When the sample was reheated to 70 °C, approximately 90% of the deformed strain was recovered. Figure 5B also demonstrates that shape recovery decreased with moisture content of the PHP film. The wet-state PHP had low shape fixity and shape recovery, which indicated that excess water molecules might destroy the original hydrogen bond. As shown in Figure 5C, the sample was first subjected to thermal orientation by heating it to its crystal melting temperature (80 °C). The new shape was deformed by an external force and then cooled to −10 °C to fix the new shape. The temporary shape was gradually recovered upon heating, and the initial shape was completely recovered thereafter within 150 s. Figure 5D demonstrates that the pentacle membrane of the PHA-based polyurethane was deformed with water at a high rate at room temperature in 5 s. The above results show that the PHA-based polyurethane samples had the water/thermal dual-responsive shape-memory properties.

**Figure 6:**
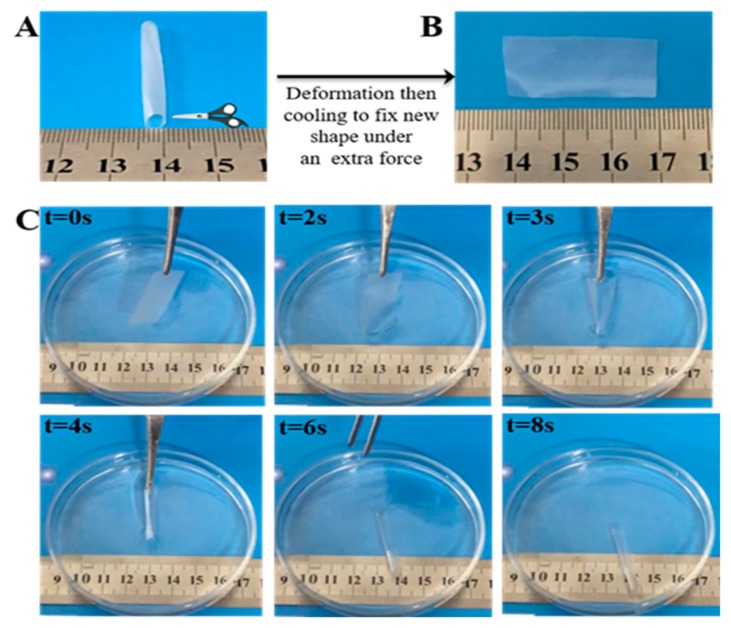
(**A**) Original shape of the PHP tube. (**B**) The tube was cut off and fixed into temporary planar shapes at −10 °C. (**C**) Time sequence of photographs showing self-folding of PHP films. The film was initially spread without water (*t* = 0), and then immersed in PBS (*t* = 2–4 s), causing rapid curling. The permanent shapes (at 37 °C in PBS) of the PHP tube in water resulted in instantaneous entangling to its original shape (*t* = 6–8 s).

Figure 6 shows the shape-morphing applications of PHP. Results showed that the temporary plane film could be simply changed to a permanent tube by instantaneously immersing it in a simulated body fluid solution. Noticeably, in all these processes, the tubes could restore to their original shapes in 10 s, demonstrating a rapid recovery process. Heterogeneous structures based on PHA-based polyurethane evolve into three-dimensional (3D) shapes (blood vessel or tissue regeneration) from 2D-structured sheets. The self-folding of the structure is controlled by the water effect. Most importantly, the shape recovery of the PHP film was rapid. The deformation process was completed within several seconds upon immersion in PBS at 37 °C. The porous scaffold of the PHP could also be self-expanding by immersion in simulated body fluid for a short time (see Figure S1). This special, rapid, and water-responsive shape-memory of our material may find important applications such as water-activated smart artificial blood vessels and controllable release devices. Desirable shape-memory properties of the shape-morphing scaffolds render them feasible for potential 3D endothelialization.

### 3.5. Mechanism of Water-Thermal Response Properties

**Figure 7:**
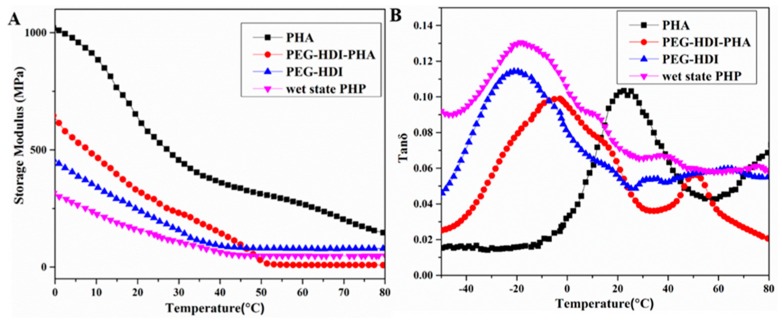
Dynamical mechanical analysis of PHA, PEG–HDI, and PEG–HDI–PHA for (**A**) storage modulus and (**B**) tan δ.

To investigatethe mechanism underlying the water–thermal response of PHA-based polyurethane, a dynamical mechanical analysis was performed, as shown in Figure 7. The thermo-mechanical properties of PHA, PEG–HDI, PEG–HDI–PHA and PHP in wet state films were established using dynamic mechanical thermal analysis. Figure 7A shows the variation in the tensile storage modulus (E’) as a function of temperature. The E’ of the samples differed with temperature. For example, at 30 °C, the E’ of PEG–HDI was only 200 MPa, whereas it increased to about 300 MPa for PHP, demonstrating a two-fold enhancement that resulted from PHA in the polymer matrix. Furthermore, the curves of loss factor (tan δ) vs. temperature (Figure 7B) showed that the intensity of the tan δ peak decreased clearly with the PHA content in the polymer matrix, indicating the interaction of PHA-diol with NCO-terminated PEG, which restricts the segmental mobility of the polymer chains. To evaluate the effect of water absorption on PHP, the storage modulus of the wetted film was also tested. The modulus of the film decreased and the intensity of the tan δ peak increased after water absorption. The significant decrease in modulus upon wetting in water can be because of the impact of water on the film, causing reduction in rigidity.

**Figure 8:**
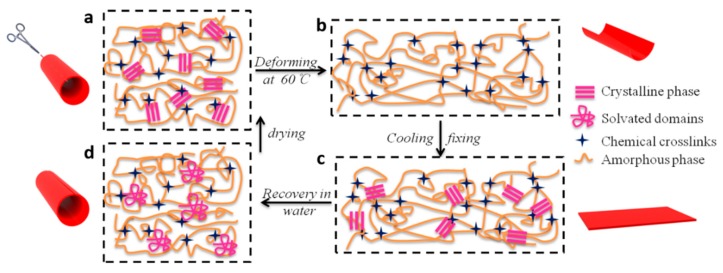
Illustration showing the mechanism underlying the shape-memory transition of PHP tubes (**a**) the dry original shape, (**b**) the heating and deforming state, (**c**) the temporary shape and (**d**) the wet original shape.

The mechanism underlying the water-triggered shape-memory performance of our PHP film is schematically shown in Figure 8. Figure 8 illustrates the micro-structural changes during the shape-memory process, wherein (a) to (b) represents deformation, (b) to (c) represents fixing, (c) to (d) denotes immersion in water, and (d) to (a) represents the drying process. FT-IR, XRD and DSC results showed that hydrogen bonding and partially crystallized zone exist in the molecular structure of PHP polymer. Many hydrogen bonds are formed because of the presence of abundant hydroxyl groups or urethane groups in the PHP molecular structure. There exists a partly thermo dynamic crystallization via hydrogen bonding. The formation of crystalline phase is favorable for fixing the material’s original shape. When the temperature is higher than T_m_, the polymer chains can move and the original shape (tubule) will be deforming.The crystalline phase of PHP can transform into the amorphous phase upon heating (Figure 8a,b). The desired temporary shape (flake) is designed under the external forces and cooling. The crystalline phase can be rebuilt after applying additional force and cooling (Figure 8b,c). Upon wetting in water, the water molecules can rapidly diffuse into the films. During this process, competitive hydrogen bonds between water molecules and hydroxyl groups on PHP can be formed, which destroys the crystalline zone and results in the appearance of solvated domains between the PHP molecular chains. Thus, the original hydrogen bond is disrupted after wetting with water, and the modulus of the PHP film is reduced. Under this condition, the film becomes soft and can be easily restored to original shape without external force (Figure 8c,d). The absorbed water is removed via drying. The hydrogen bonds and crystalline phase between PHP molecule chains can be rebuilt (Figure 8a,d).

## 4. Conclusions

This study reports the synthesis and characterizations of bio-based shape-memory polyurethane with dual-responsive SMEs from PHA. After introduction of the PHA and PEG soft segments, the obtained PHP showed good thermal-responsive and water-responsive shape-memory behaviors. Furthermore, they possessed excellent mechanical performance, with superior stretchability and compressibility. Accordingly, PHP was easily fabricated into membranes, tubes, and porous scaffolds for several potential applications. Finally, the fast water-responsive recovery was due to dissociation of hydrogen bonding upon wetting, resulting in the destruction of the crystalline soft segment. Owing to its excellent water-responsive SME, PHP shows potential shape-morphing applications in tissue engineering.

## Supplementary Materials

The following are available online at https://www.mdpi.com/2073-4360/11/6/1030/s1, Figure S1: (A) The original shape (*d* = 18 mm, *t* = 0 s), (B) The temporary shape after compressing under external force (*d* = 10 mm), (C) recover after absorbing simulated body fluid with different time, (D) The model of porous scaffolds self-expanding response, Table S1: Identification of characteristic IR frequencies of PHA-based polyurethane.
