# Supplementary material for: Development of Polyhydroxyalkanoate-Based Polyurethane with Water-Thermal Response Shape-Memory Behavior as New 3D Elastomers Scaffolds
Source: Polymers (Basel). 2019 Jun 11;11(6):1030. doi: 10.3390/polym11061030 (PMC6631955; doi:10.3390/polym11061030)
Supplement: Supplementary file 1 [file polymers-11-01030-s001.pdf]

Development of polyhydroxyalkanoate-based polyurethane with water-thermal response shape-memory behavior as new 3D elastomers scaffolds

Cai Wang[1,2], Han Wang[1], Faxing Zou[1], Shaojun Chen[1]* and Yiping Wang[2]*

1 Guangdong Research Center for Interfacial Engineering of Functional Materials, Shenzhen Key Laboratory of Polymer Science and Technology, Shenzhen Key Laboratory of Special Functional Materials, Nanshan District Key Lab for Biopolymers and Safety Evaluation, College of Materials Science and Engineering, Shenzhen University, Shenzhen 518060, China

2 Key Laboratory of Optoelectronic Devices and Systems of Ministry of Education and Guangdong Province, College of Optoelectronic Engineering, Shenzhen University, Shenzhen 518060, China

* Correspondence: chensj@szu.edu.cn; ypwang@szu.edu.cn

Experimental section

Preparation of PHP porous scaffolds

The particulate leaching methodwas used to prepare the PHP porous scaffolds.Briefly, a polymer solution was prepared by mixing a PHApellet with HDI and PEG solution. The solution was then stirred atroom temperature for 4 h. Next, NaCl particles ranging indiameter about 150um (polymer/NaCl =1/20 (w/w))were added. The mixture was packed into Petri dishes, creating cylindrical molds that were 15 mm in diameter and 15mm in thickness. These molds were placed in a ventilationhood overnight to allow solvent evaporation. After evaporation, to leach out the salt particles, the constructswere immersed in deionized (DI) water for 72 h, withrepeated changes of the DI water every 4 h. The scaffoldswere vacuum-dried for 24 h.The resultant PHP porous scaffolds exhibited highlyinterconnected porous networks.

Supplementary tables and figures

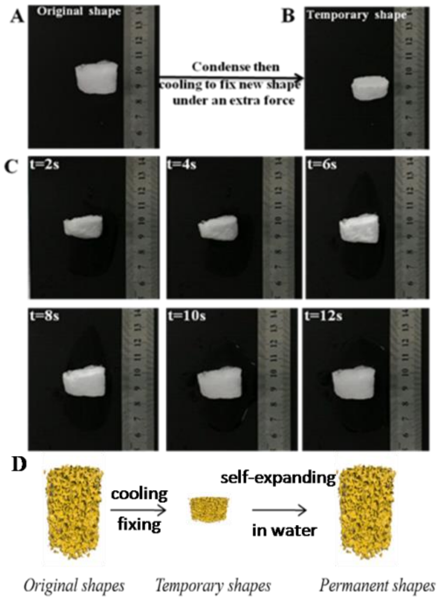

Fig.S1 (A) The original shape(d=18mm,t=0s), (B) The temporary shape after compressing under external force(d=10mm),(C) recover after absorbing simulated body fluid with different time,(D) The model of porous scaffolds self-expanding response.

Table 1 Identification of characteristic IR frequencies of PHA-basedpolyurethane

Groups	Wavenumber(cm^{-1})	Assignment
-N-H	3341	Hydrogen bonded N-H vibration band
$-CH_2$	2915	Methylene stretching vibration modes(asym)
$-CH_2$	2856	Methylene stretching vibration modes(sym)
-C=O	1721	Ester stretching vibration modes
-C=O	1622	Hydrogen bonded urethane stretching vibration
-CH2	1578	C-H bending vibration
-CH3	1456	C-H bending vibration(asym)
-C-N	1441	C-N stretching vibration
-CH3	1356	C-H bending vibration(sym)
-C-O-C	1253	Ester stretching vibration (crystalline)
-C-O-C	1096	Stretching vibration mode
-C-O-C	983	Ester stretching vibration
-O-C-C	840	Stretching vibration mode
-O-C-N	636	Stretching vibration mode
